# Causes of hospitalization and predictors of HIV-associated mortality at the main referral hospital in Sierra Leone: a prospective study

**DOI:** 10.1186/s12889-019-7614-3

**Published:** 2019-10-21

**Authors:** Sulaiman Lakoh, Darlinda F. Jiba, Joseph E. Kanu, Eva Poveda, Angel Salgado-Barreira, Foday Sahr, Momodu Sesay, Gibrilla F. Deen, Tom Sesay, Wadzani Gashau, Robert A. Salata, George A. Yendewa

**Affiliations:** 1 College of Medicine and Allied Health Sciences, University of Sierra Leone, Freetown, Sierra Leone; 2 Department of Medicine, University of Sierra Leone Teaching Hospitals Complex, Freetown, Sierra Leone; 3 Group of Virology and Pathogenesis, Galicia Sur Health Research Institute (IIS Galicia Sur)-Complexo Hospitalario Universitario de Vigo, SERGAS-UVigo, Vigo, Spain; 4 Methodology and Statistics Unit, Galicia Sur Health Research Institute (IIS Galicia Sur)-Complexo Hospitalario Universitario de Vigo, SERGAS-UVigo, Vigo, Spain; 5 National HIV/AIDS Secretariat, Freetown, Sierra Leone; 6 National AIDS Control Programme, Ministry of Health and Sanitation, Freetown, Sierra Leone; 7 Department of Medicine, Case Western Reserve University School of Medicine, Cleveland, OH USA; 8 Division of Infectious Diseases and HIV Medicine, University Hospitals Cleveland Medical Center, Cleveland, OH USA; 9 Johns Hopkins Bloomberg School of Public Health, Baltimore, MD USA

**Keywords:** HIV, Diagnosis, Mortality, In-hospital, Sierra Leone

## Abstract

**Background:**

HIV infection is a growing public health problem in Sierra Leone and the wider West Africa region. The countrywide HIV prevalence was estimated at 1.7% (67,000 people), with less than 30% receiving life-saving ART in 2016. Thus, HIV-infected patients tend to present to health facilities late, with high mortality risk.

**Methods:**

We conducted a prospective study of HIV inpatients aged ≥15 years at Connaught Hospital in Freetown—the main referral hospital in Sierra Leone—from July through September 2017, to assess associated factors and predictors of HIV-related mortality.

**Results:**

One hundred seventy-three HIV inpatients were included, accounting for 14.2% (173/1221) of all hospital admissions during the study period. The majority were female (59.5%, 70/173), median age was 34 years, with 51.4% (89/173) of them diagnosed with HIV infection for the first time during the current hospitalization. The most common admitting diagnoses were anemia (48%, 84/173), tuberculosis (24.3%, 42/173), pneumonia (17.3%, 30/173) and diarrheal illness (15.0%, 26/173). CD4 count was obtained in 64.7% (112/173) of patients, with median value of 87 cells/μL (IQR 25–266), and was further staged as severe immunosuppression: CD4 < 100 cells/μL (50%, 56/112); AIDS: CD4 < 200 cells/μL (69.6%, 78/112); and late-stage HIV disease: CD4 < 350 cells/μL (83%, 93/112). Fifty-two patients (30.1%, 52/173) died during hospitalization, 23% (12/52) of them within the first week. The leading causes of death were anemia (23.1%, 12/52), pneumonia (19.2%, 10/52), diarrheal illness (15.4%, 8/52) and tuberculosis (13.6%, 7/52). Neurological symptoms, i.e., loss of consciousness (*p* = 0.04) and focal limb weakness (*p* = 0.04); alcohol use (*p* = 0.01); jaundice (*p* = 0.02); cerebral toxoplasmosis (*p* = 0.01); and tuberculosis (*p* = 0.04) were significantly associated with mortality; however, only jaundice (AOR 0.11, 95% CI [0.02–0.65]; *p* = 0.01) emerged as an independent predictor of mortality.

**Conclusion:**

HIV-infected patients account for a substantial proportion of admissions at Connaught Hospital, with a high morbidity and in-hospital mortality burden. These findings necessitate the implementation of specific measures to enhance early HIV diagnosis and expand treatment access to all HIV-infected patients in Sierra Leone.

## Background

Despite the global roll-out of antiretroviral therapy (ART), high in-hospital mortality associated with human immunodeficiency virus (HIV) infection remains a major problem in HIV care in resource-limited settings. The United Nations Joint Programme on HIV and AIDS (UNAIDS) 2017 Report has estimated that since the beginning of the global HIV pandemic in 1981, 35.4 million acquired immunodeficiency syndrome (AIDS)-related deaths have occurred worldwide—with 940,000 deaths recorded in 2017 alone –despite death rates actually declining since 2005 [1]. A disproportionately high number of the global total deaths (70%) occurred in sub-Saharan Africa, primarily in the high burden countries where the majority of HIV-infected people reside [1]. Of note, the proportion of deaths reported from the World Health Organization (WHO) region of West and Central Africa was particularly striking. Although countries in this WHO region have consistently reported low HIV seroprevalence rates for many years (generally < 4%) [2], West and Central Africa accounted for 30% (280,000) of the global mortality in 2017 [1], highlighting a regional public health problem of growing magnitude and importance.

Several studies from West Africa have previously assessed the prevalence and associated factors of AIDS-related in-hospital mortality. A review of 207 HIV-infected patients admitted at a northern Nigerian tertiary health center recorded a 31.9% mortality rate (66 deaths) in 2012 [3]. Similarly, another study from Ghana (*n* = 547) reported an AIDS-associated mortality rate of 40.6% (222/547) during 2012–2013, with 55.9% (124/222) of those deaths occurring in the first week of hospitalization [4]. The most comprehensive study to date was conducted by the West Africa International epidemiological Database to Evaluate AIDS (IeDEA) group in 2010 and it included a total of 823 participants at clinical sites in 5 West African countries (Benin, Burkina Faso, Cote d’Ivoire, Mali and Senegal). This study recorded an in-hospital mortality rate of 38% [5]; however, given that it was conducted almost a decade ago before HIV services were scaled-up, the findings may not be reflective of current trends—hence, the need for an update from countries in the region.

In Sierra Leone, the HIV epidemic has steadily expanded ever since the first 10 cases were reported among commercial sex workers in 1987—and has been fueled, in part, by a severely under-resourced healthcare system, a decade long civil war (1991–2000) and disruptions in HIV care due to the recent Ebola epidemic (2014–2016) [6]. The latest countrywide HIV seroprelavance rate was estimated at 1.7% in 2016, representing a total of 67,000 HIV-infected people, with less than 30% receiving ART [7]. Over a 6-year period (2010–2016), the country saw a 7% increase in new infections and a 6% rise in AIDS-related deaths [7]. In a cross-sectional study of newly diagnosed HIV-infected outpatients at the HIV clinic at Connaught Hospital in Freetown (the main referral hospital in Sierra Leone), we previously documented a high prevalence (75%, *n* = 155) of late stage HIV presentation among newly diagnosed patients, defined as CD4 < 350 and/or the presence of an AIDS-defining illness at diagnosis [8]. However, few studies have examined the disease progression patterns and clinical outcomes of HIV-infected patients in this West African country.

In this prospective study, we assessed the prevalence, clinical presentation, associated factors and predictors of in-hospital HIV-related mortality among adult HIV-infected patients admitted to Connaught Hospital in Freetown.

## Methods

### Study population and setting

A total of 173 HIV-infected patients aged ≥15 years who were admitted at Connaught Hospital in Freetown were consecutively selected and included in the study from July through September 2017. Connaught Hospital is a 300-bed academic facility located in Freetown in Sierra Leone. It is also the main referral hospital for the management of medical and surgical cases in the country and is affiliated with the College of Medicine and Allied Health Sciences of the University of Sierra Leone, the country’s only medical school. It has 10 wards (5 surgical, 4 medical, 1 pediatric), 1 intensive care unit, 2 accident and emergency wards (medical and surgical) and 2 private wards (for male and female patients, respectively). It is also the site of the largest HIV clinic and treatment center in the country. The facility offers both outpatient and in-patient services including HIV Counselling and Testing Services (HCTS), Prevention of Mother to Child Transmission (PMTCT) services, Antiretroviral Treatment (ART), and treatment of opportunistic infections.

### Clinical data collection and ethical considerations

Demographic and clinical data were recorded. Informed written consent from participants aged ≥18 years and written parental consent was obtained for patients aged 15–18 years prior to enrollment in the study. All patient personal information were de-indentified before data were transferred into a password protected spreadsheet accessible only to study personnel. Ethical approval for the study was obtained from the Sierra Leone Ethics Scientific and Research Committee.

### Diagnoses and definitions

The fourth-generation rapid test by SD Bioline HIV-1/2 3.0 (Standard Diagnostics Inc) was used for HIV testing. CD4 cell count was determined using the Alere Pima™ Analyzer (Abbott), a point-of-care testing platform with comparable performance to flow cytometry-based methods and validated in resource-limited settings [9]. Tuberculosis was confirmed using the GeneXpert MTB/RIF assay (Cepheid) and/or sputum acid fast bacilli (AFB) smear, with compatible chest radiographic findings. Cerebral toxoplasmosis was diagnosed clinically based on the presence of focal neurologic deficit(s) with/without features of encephalitis and the presence of ring-enhancing cranial lesions on CT imaging. Other AIDS-defining illnesses (ADIs) such as cryptococcal meningitis, pneumocystis pneumonia, Kaposi sarcoma, and esophageal candidiasis were diagnosed on the basis of compatible clinical findings. Coinfections with malaria and hepatitis B virus (HBV) were confirmed with the appropriate serological tests in the setting of compatible physical and/or clinical findings. Sepsis was defined in accordance with the Third International Consensus Definitions for Sepsis, which considers a quick sequential organ failure assessment score (qSOFA score) ≥ 2 in the setting of a suspected or confirmed microbiologic etiology as diagnostic of sepsis [10]. Anemia was defined as hemoglobin concentration of < 11.0 g/dL for the adult non-pregnant population, which is the lower limit of the reference range at the Connaught Hospital Laboratory. Alcohol use was defined as consuming > 20 g or 2 drinks of an alcoholic beverage daily, while illicit drug use was defined as using any quantity of any of marijuana, cocaine or injected heroine in the last 30 days.

In all instances, assiduous attempts were made by the three physicians who collected the clinical data to identify and confirm the admitting diagnoses and causes of death on the basis of test results, diagnostic criteria and other available medical records. In the absence of these, compatible physical and/or clinical findings and other supporting clinical evidence were used to arrive at a consensus presumptive diagnosis or cause of death.

### Statistical analysis

All statistical analyses were performed using the software package SPSS Statistics Version 25.0 (Armonk, NY: IBM Corp). Categorical variables were reported as frequency (percentage) and compared using the Pearson’s chi-squared (χ^2^) test. Continuous variables were recorded as median (interquartile range) and compared using the nonparametric independent samples Mann-Whitney U test or exact Fischer test, where appropriate. Associations were tested between in-hospital mortality and the following variables in the unadjusted univariate analysis: age, gender, specific risk factors (smoking, alcohol use, illicit drug use), CD4 count, WHO clinical staging, admitting diagnosis, ART status, and length of hospital stay in the unadjusted univariate analysis. A logistic regression model was used to determine the independent predictors of in-hospital mortality in the multivariate analysis. Other than age, gender and ART status which were a priori included, only variables that achieved *p* < 0.2 in the univariate analysis were included in the multivariate regression analysis. To determine the odds of mortality with older age, age was further stratified into younger (15–44 years) and middle/older (≥ 45 years) categories before inclusion in the model. In all analyses, *p* < 0.05 was considered statistically significant.

## Results

During the study period, a total of 1221 patients were admitted to the various wards at Connaught Hospital, representing an estimated bed occupancy rate of 95.0%. About 32.9% of all hospitalized patients (402/1221) were admitted to the medical wards. Thus, the 173 HIV-infected individuals enrolled in our study represented 43.0% (173/402) of patients admitted to the medical wards and 14.2% (173/1221) of all hospital admissions during the study period.

### Baseline characteristics of study participants

Table 1 presents the baseline characteristics of the study participants. Of a total of 173 HIV-infected patients who were included in the study, the majority were female (59.5%, 103/173), with a median age of 34 years (IQR 28–44) and a median length of hospital stay of 12 days (IQR 8–17). Most participants had some form of education (70.5%, 122/173), with the secondary level being the most frequently attained (50.9%, 88/173). About two-thirds reported being single. The proportion of patients who admitted to smoking, alcohol use, and illicit drug use were 20.2% (35/173), 22.5% (39/173) and 5.2% (9/173), respectively.

**Table 1:** Baseline characteristics of newly admitted HIV-patients at Connaught Hospital

Characteristics	*N (%)* 173 (100%)
Gender	
Male	70 (40.5)
Female	103 (59.5)
Age, *years*	
Median, IQR	34 (28–44)
15–24	25 (14.5)
25–34	68 (39.3)
35–44	40 (23.1)
45–54	26 (15.0)
55–64	9 (5.2)
> 64	5 (2.9)
Educational level	
None	51 (29.5)
Primary	19 (11.0)
Secondary	88 (50.9)
Tertiary	15 (8.7)
Marital Status	
Married	52 (30.1)
Single	116 (67.0)
Unknown	5 (2.9)
Occupation	
Trader/Business	68 (39.3)
Student	19 (11.0)
Unemployed	4 (2.3)
Other	82 (47.4)
Risk factors	
Smoking	35 (20.2)
Alcohol use	39 (22.5)
Illicit drugs	9 (5.2)
HIV status	
Known before admission	
< 12 months	21 (12.2)
> 12 months	53 (30.6)
Diagnosed during hospitalization	89 (51.4)
Unknown	10 (5.8)
ART regimens, *N* = 64 (100%)	
TDF + 3TC + EFV	54 (84.4)
AZT + 3TC + NVP	7 (10.9)
AZT + 3TC + EFV	1 (1.6)
AZT + 3TC + LPV/r	2 (3.1)
CD4 count, cells/μL, *N* = 112 (100%)	
Median, IQR	87 (25–266)
< 100	56 (32.3)
100–199	22 (12.7)
200–349	15 (8.7)
≥ 350	19 (11.0)
Not obtained	61 (35.3)
WHO clinical staging	
Stage 1	1 (0.6)
Stage 2	28 (16.1)
Stage 3	115 (66.5)
Stage 4	29 (16.8)

*IQR* interquartile range, *AZT* zidovudine, *TDF* tenofovir disoproxil fumarate, *3TC* lamivudine, *EFV* efavirenz, *NVP* nevirapine, *LPV/r* lopinavir/ritonavir

Over half of all study participants (51.4%, 89/173) were diagnosed with HIV infection for the first time during the hospitalization; the remainder (48.6%, 84/173) had a known history of HIV infection prior to hospitalization, with 25% (21/84) of them having been first diagnosed within the preceding 12 months. About 6% (10/173) could not confirm whether they had been previously diagnosed. The majority were infected with HIV-1 type (96.0%, 166/173), while 4% (7/173) of patients had HIV test results that were indeterminate for virus type. No cases of HIV-2 mono-infection or HIV-1/HIV-2 dual infection were detected.

In total, only 37.0% (64/173) of study participants were receiving ART during the hospital stay. The most common regimen was tenofovir/lamivudine/efavirenz (84.4%, 54/64); the remaining patients were on zidovudine/lamivudine/nevirapine (10.9%, 7/64); zidovudine/lamivudine/lopinavir/ritonavir (3.1%, 2/64); and zidovudine/lamivudine/efavirenz (1.6%, 1/64). Ninety-six percent (166/173) of all patients were receiving cotrimoxazole prophylaxis during hospital stay, in line with current WHO recommendations for treatment of HIV infection in resource-limited settings [11].

### Clinical presentation and admitting diagnoses

Table 2 shows the clinical presentation (signs and symptoms) and admitting diagnoses of the study participants. The majority (94.2%, 163/173) reported constitutional symptoms; other symptoms by systems included cardiopulmonary (63.6%, 110/173), gastrointestinal (54.9%, 95/173), and neurological (37.0%, 64/173) and genitourinary (15.0%, 26/173). The most common symptoms were fever (77.5%, 163/173), cough (56.1%, 110/173), weight loss (53.8%, 134/173), generalized malaise (53.2%, 92/173), anorexia (38.7%, 67/173), and diarrhea (38.2%, 95/173).

**Table 2:** Presenting signs and symptoms and admitting diagnoses of HIV in-patients

Presenting Signs, Symptoms and Diagnoses	*N (%)* 173 (100)
Constitutional	163 (94.2)
Fever	134 (77.5)
Weight loss	93 (53.8)
Malaise	92 (53.2)
Anorexia	67 (38.7)
Night sweats	20 (11.6)
Rash	17 (9.8)
Neurological	64 (37.0)
Headache	33 (19.1)
Seizure	2 (1.2)
Loss of consciousness	23 (13.3)
Neck stiffness	5 (2.9)
Limb weakness	8 (4.6)
Cardiopulmonary	110 (63.6)
Cough	97 (56.1)
Chest pain	39 (22.5)
Dyspnea	29 (16.8)
Hemoptysis	2 (1.2)
Gastrointestinal	95 (54.9)
Diarrhea	67 (38.2)
Abdominal pain	34 (19.7)
Nausea/vomiting	33 (19.1)
Jaundice	7 (4.0)
Ascites	9 (5.2)
Hepatosplenomegaly	15 (8.7)
Odynophagia	11 (6.4)
Genitourinary	26 (15.0)
Frequency	9 (34.6)
Dysuria	4 (2.3)
Urgency	3 (1.7)
Hematuria	2 (1.2)
Genital ulcers	3 (1.7)
Admitting diagnoses	
Anemia	83 (48.0)
Primary diagnosis	26 (15.0)
In association with other conditions	57 (33.0)
Tuberculosis	42 (24.3)
Pneumonia	30 (17.3)
Diarrheal illness	26 (15.0)
Malaria	11 (6.4)
Cerebral toxoplasmosis	9 (5.2)
Sepsis	6 (3.5)
Decompensated liver cirrhosis	5 (2.9)
Gastroenteritis	5 (2.9)
Disseminated Kaposi sarcoma	4 (2.3)
Esophageal candidiasis	4 (2.3)
Cryptococcal meningitis	3 (1.7)
Pneumocystis pneumonia	3 (1.7)
Drug-induced hepatotoxicity	1 (0.6)
*Salmonella typhi* infection	1 (0.6)

The most common admitting diagnosis was anemia (48.0%, 83/173 in total; in 15.0% (26/173) of cases as the primary admitting diagnosis and in 33% (57/173) of cases associated with other admitting diagnosis), followed by tuberculosis (24.3%, 42/173), pneumonia (17.3%, 30/173), diarrheal illness (15.0%, 26/173), malaria (6.4%, 11/173), cerebral toxoplasmosis (5.2%, 9/173), sepsis (3.5%, 6/173), hepatitis B virus (HBV)-related decompensated liver cirrhosis (2.9%, 5/173), gastroenteritis (2.9%, 5/173), disseminated Kaposi sarcoma (2.3%, 4/173), esophageal candidiasis (2.3%, 4/173) and cryptococcal meningitis (1.7%, 3/173).

### Immunological profile and WHO clinical staging

The immunological profile of participants was assessed (Table 1). The CD4 count was obtained for only 64.7% (112/173) of patients during the hospital stay. The median CD4 count was 87 cells/μL (IQR 25–266). The distribution of patients based on CD4 count was further stratified as follows: CD4 < 100cells/μL, i.e., severe immunosuppression 50% (56/112); CD4 < 200 cells/μL, i.e., AIDS 69.6% (78/112); and CD4 < 350 cells/μL, i.e., late-stage HIV disease 83.0% (93/112).

The WHO staging was obtained for all patients, with the majority (83.2%, 114/173) being classified as either WHO stage 3 or 4 (Table 1).

### Distribution of AIDS-defining conditions

The distribution of AIDS-defining conditions diagnosed (not mutually exclusive) was as follows: tuberculosis (24.3%, 42/173), cerebral toxoplasmosis (5.2%, 9/173), esophageal candidiasis (2.3%, 4/173), Kaposi sarcoma (2.3%, 4/173), Pneumocystis pneumonia (1.7%, 3/173), cryptococcal meningitis (1.7%, 3/173), and *Salmonella typhi* infection (0.6%, 1/173) (Table 2).

### Causes of death

A total of 52 deaths occurred during hospitalization, yielding a HIV-associated in-hospital mortality rate of 30.1%. About 23.1% (12/52) of all deaths occurred within the first 7 days of hospitalization. The leading causes of death were anemia (23.1%, 12/52), pneumonia (19.2%, 10/52), diarrheal illness (15.4%, 8/52) and tuberculosis (13.6%, 7/52). Table 3 displays the other causes of death stratified along ART-usage, given the low proportion (37%) of patients who were on life-saving ART prior to hospitalization.

**Table 3:** Causes of death in hospitalized HIV-infected patients related to ART status

Cause of death	Total *N* = 52 (100%)	ART status		*P* value
		No *N* = 36 (100%)	Yes *N* = 16 (100%)	
Anemia	12 (23.1)	10 (27.8)	2 (12.5)	0.17
Pneumonia	10 (19.2)	7 (19.4)	3 (18.8)	0.95
Diarrheal illness	8 (15.4)	3 (8.3)	5 (31.3)	**0.04**
Tuberculosis	7 (13.6)	4 (11.0)	3 (18.8)	0.46
Cerebral toxoplasmosis	6 (11.5)	5 (13.9)	1 (6.2)	0.43
Decompensated cirrhosis	3 (5.8)	2 (5.6)	1 (6.2)	0.92
Pneumocystis pneumonia	1 (1.9)	1 (2.8)	–	0.50
Malaria	1 (1.9)	1 (2.8)	–	0.50
Bacterial meningitis	1 (1.9)	1 (2.8)	–	0.50
Sepsis	1 (1.9)	–	1 (6.2)	0.13
Drug-induced hepatotoxicity	1 (1.9)	1 (2.8)	–	0.50
Stroke	1 (1.9)	1 (2.8)	–	0.50

### Associated factors and predictors of in-hospital mortality

In the univariate analysis, alcohol use (34.6% versus 14.4%, *p* = 0.01); presence of jaundice (9.6% versus 1.7%, *p* = 0.02); presenting with limb weakness (9.6% versus 2.5%, *p* = 0.04); loss of consciousness (9.6% versus 2.5%, *p* = 0.04); tuberculosis (13.5% versus 28.1%, *p* = 0.04); or cerebral toxoplasmosis (11.5% versus 2.5%, *p* = 0.01) were significantly associated with in-hospital mortality (Table 4). No association was observed based on age, gender, CD4 count, WHO clinical stage, ART status or length of hospital stay. In the multivariate logistic regression analysis, younger age (15–44 years), middle/older age (≥ 45 years), gender, ART status and variables with *p* < 0.2 in the univariate analysis were included. After adjusting the model, jaundice (AOR 0.11, 95% CI [0.02–0.62]; *p* = 0.01) was identified as the only independent predictor of in-hospital mortality (Table 5).

**Table 4:** Factors associated with HIV-related in-hospital mortality

Characteristics	Survival status		*P* value
	Died *N* = 52 (100%)	Discharged alive *N* = 121 (100%)	
Age, *years*			
Median (IQR)	37 (29–45)	33 (28–42)	0.47
Gender, *N%*			
Male	20 (38.5)	50 (41.3)	0.73
Female	32 (61.5)	71 (58.7)	
Risk factors, *N%*			
Smoking	15 (28.8)	20 (16.5)	0.06
Alcohol use	18 (34.6)	21 (17.4)	**0.01**
Illicit drug use	5 (13.5)	4 (3.3)	0.09
CD4 count, *cells/μL*			
Median (IQR)	70 (23–263)	101 (29–278)	0.61
Less than 50, *N%*	12 (23.1)	23 (19.0)	0.82
WHO Clinical Staging, *N%*			
1 or 2	6 (11.5)	22 (18.2)	0.28
3 or 4	46 (88.5)	99 (81.8)	
Presenting signs or symptoms, *N%*			
Fever	42 (80.8)	92 (76.0)	0.49
Jaundice	5 (9.6)	2 (1.7)	**0.02**
Weight loss	30 (57.7)	63 (52.1)	0.50
Headache	11 (21.2)	22 (18.2)	0.65
Seizure	–	2 (1.7)	0.35
Limb weakness	5 (9.6)	3 (2.5)	**0.04**
Loss of consciousness	5 (9.6)	3 (2.5)	**0.04**
Admitting diagnoses, *N%*			
Anemia	25 (48.1)	58 (47.9)	0.99
Tuberculosis	7 (13.5)	34 (28.1)	**0.04**
Pneumonia	10 (19.2)	20 (16.5)	0.67
Diarrheal illness	8 (15.4)	18 (14.9)	0.93
Cerebral toxoplasmosis	6 (11.5)	3 (2.5)	**0.01**
Cryptococcal meningitis	1 (1.9)	2 (1.7)	0.90
New Diagnosis, *N%*			
Yes	29 (55.8)	60 (49.6)	0.46
No	23 (44.2)	61 (50.4)	
ART status, *N%*			
Yes	16 (30.8)	48 (39.7)	0.19
No	36 (69.2)	73 (60.3)	
Length of hospital stay, *days*			
Median	11 (8–16)	12 (9–18)	0.48

*IQR* interquartile range

**Table 5:** Predictors of in-hospital HIV-related mortality

Characteristics	Univariate		Multivariate	
	OR (95% CI)	*P* value	AOR (95% CI)	*P* value
Middle/older age (≥ 45 years)	0.97 (0.44–2.16)	0.94	0.87 (0.36–2.10)	0.76
Gender	0.89 (0.46–1.73)	0.73	1.48 (0.67–3.27)	0.33
Smoking	2.05 (0.95–4.41)	0.06	0.77 (0.25–2.37)	0.65
Alcohol use	2.52 (1.20–5.28)	**0.01**	0.36 (0.11–1.12)	0.08
Illicit drug use	3.11 (0.80–12.10)	0.09	0.44 (0.09–2.26)	0.33
Jaundice	6.33 (1.19–33.77)	**0.02**	0.11 (0.02–0.62)	**0.01**
Limb Weakness	4.18 (0.96–18.21)	**0.04**	0.47 (0.08–2.78)	0.40
Loss of consciousness	4.18 (0.96–18.21)	**0.04**	0.40 (0.08–2.12)	0.28
Cerebral toxoplasmosis	5.13 (1.23–21.38)	**0.01**	0.29 (0.05–1.48)	0.13
Tuberculosis	0.40 (0.16–0.97)	**0.03**	2.48 (0.91–6.70)	0.07
ART status	0.63 (0.32–1.26)	0.19	1.28 (0.60–2.76)	0.52

*OR* odds ratio, *AOR* adjusted odds ratio, *CI* confidence interval

## Discussions

This is the first study to assess HIV-related admissions and in-hospital mortality at the main referral health center in Sierra Leone. A high prevalence of HIV-associated in-hospital mortality (30.1%, 52/173) was observed in a cohort of 173 HIV-infected individuals who were admitted to the medical wards at Connaught Hospital in Freetown during July to September in 2017**.** About 22% (12/52) of those deaths occurred within the first week of hospitalization. The median length of hospital stay was 12 days. The in-hospital mortality rate was similar to that obtained from the northern Nigerian cohort discussed earlier [3] but lower than findings from Ghana [4] and the 2010 West Africa IeDEA cohort study [5]. This likely reflects the higher burden of HIV disease and/or local healthcare challenges in the clinical management of patients in those countries before HIV services and ART programs were scaled-up. Furthermore, over 50% (89/173) of patients were diagnosed with HIV infection for the first time during hospitalization versus 24% in the West Africa IeDEA study [5]—not a surprising finding given that even though there are high levels of HIV awareness among adults in Sierra Leone (over 94%), uptake in voluntary HIV testing has remained below 30% in the country [12].

Tuberculosis was one of the leading admitting diagnoses (24.3%, 42/173) and although it was not the leading cause of death, it accounted for a substantial proportion of deaths (13.6%, 7/52) and was significantly associated with in hospital mortality (*p* = 0.03). According to the WHO 2017 report, tuberculosis remains the most common presentation in HIV-infected individuals and is the leading cause of death among HIV-infected people worldwide [13]. It is noteworthy that an even higher proportion of deaths were accounted for by pneumonia (19.2%, 10/52) in this study. Given the predominance of atypical radiographic findings in HIV-tuberculosis coinfection [14, 15], it is possible that there may have been some overlap between the two conditions, which may have resulted in underestimation of true number of tuberculosis cases.

Anemia is a major hallmark of HIV disease progression and was associated with 48% (83/173) of admissions and 23% (12/52) of deaths. The pathogenesis of HIV-associated anemia has been well described, with a broad differential including direct myelosuppression by HIV itself leading to impaired/reduced erythropoiesis or increased hemolysis, infiltrative neoplasms, tuberculosis, malaria coinfection in endemic regions and ART/other drug-associated toxicities—most notably zidovudine use [16, 17]. Of note, about 16% (10/64) of our study participants receiving ART were on a zidovudine-containing regimen. Severe anemia was found to be an independent predictor of early mortality in HIV infection in multiple studies [3, 18, 19] but was not significantly associated with mortality in this study.

The immunological profile of our study participants as determined by the CD4 count distribution and WHO clinical staging highlight the growing problem of late-stage HIV presentation felt most keenly in sub-Saharan African countries [8, 20–23]. In the study participants for whom CD4 count was obtained, 83% (93/112) met the criteria for late-stage HIV disease (i.e., CD4 < 350 cells/μL), coinciding with the 83.2% (144/173) of patients that were also clinically classified as either WHO stage 3 or 4. Although this suggests that WHO staging could be a reasonably accurate and cost-effective means of classifying HIV disease severity that can be applied in rural areas in Sierra Leone where CD4 cell count testing capability may not be readily, a dedicated study assessing the specificity and sensitivity of this method is warranted. Furthermore, nearly 70% (78/112) met the criteria for AIDS (i.e., CD4 < 200 cells/μL) and over 50% (56/112) were severely immunosuppressed (i.e., CD4 < 100 cells/μL). Recent estimates from elsewhere in sub-Saharan Africa have also reported a high prevalence of advanced HIV disease among hospitalized HIV patients—83.7 and 97.3%, in Kenya and the Democratic Republic of Congo, respectively [24]. Late stage HIV diagnosis has been associated with higher morbidity and mortality [25, 26] and escalated treatment costs in an already overburdened healthcare system [27]. In our study however, mortality was not associated with late-stage HIV presentation, likely due to the sample size of the study.

Other factors significantly associated with in-hospitality were, most prominently, the presence of neurological symptoms—i.e., loss of consciousness (*p* = 0.04) and focal limb weakness (*p* = 0.04), cerebral toxoplasmosis (*p* = 0.01), alcohol use (*p* = 0.01) and jaundice (*p* = 0.02); however, only jaundice emerged as an independent predictor of in-hospital mortality (AOR 0.11, 95% CI [0.02–0.65]; *p* = 0.01) (Table 5). The etiologies of jaundice in HIV disease span a broad spectrum of conditions. In hyper-endemic countries such as Sierra Leone, jaundice is most often associated with malaria or acute HBV infection, but may also be a prominent clinical feature of alcoholic liver disease, drug-induced hepatotoxicity, tuberculosis-associated granulomatous hepatitis, AIDS cholangiopathy, hepatocellular carcinoma, schistosomiasis, hemangioma, and hepatic adenoma [28, 29]. Several observational studies have shown that HIV coinfection with HBV can lead to rapid more progression to AIDS [30, 31], adverse complications such as the immune reconstitution syndrome on ART initiation [32, 33], and faster onset of cirrhotic liver disease and hepatocellular carcinoma [33, 34]. Malaria or HBV infection was present in less than 10% (16/173) of our study participants; however, screening was only undertaken in select patients who exhibited suggestive signs or symptoms of liver disease; it is therefore conceivable that the true prevalence of asymptomatic malaria and viral hepatitis may have been much higher in our cohort. Of note, we recently observed a high prevalence of serological markers of chronic HBV infection, i.e., HBsAg (22%, 38/175) and prior exposure, i.e., total anti-HBc (83%, 175/211) in a separate cohort of 211 HIV-infected outpatients at Connaught Hospital [35]. In another cohort of HIV infected patients, we also recorded a high prevalence of pretreatment HIV drug resistance (14.2%) to nucleoside/nucleotide reverse transcriptase inhibitors (NRTIs) among ART-naïve patients (*n* = 64); among the ART-exposed patients (*n* = 151), the prevalence acquired HIV drug resistance to NRTIs was 6-fold higher at 85.2% [36]. The high levels HIV/HBV co-infection and high cross-resistance rates to NRTIs which are integral treatment regimens for both infections may have far-reaching implications for disease outcomes in HBV hyper-endemic countries such as Sierra Leone and therefore warrant further investigation.

Our study had several limitations, including the small sample size, potentially making the findings not readily generalizable. There was potential selection bias in this study, as patients at the national referral health center would be expected to more severely ill and therefore possibly at higher risk of mortality. Determining admission diagnoses and causes of death were major challenges, given that advanced diagnostic tools which are not readily available in resource-limited settings are needed to determine the etiology of certain infections and AIDS-defining conditions and thus, most diagnoses were presumptive. Incompleteness of the CD4 cell count data was another limitation; this is because the study relied on the availability of point-of-care testing data per standard of care. Furthermore, the study did not track patients in non-medical wards, which may have underestimated the true prevalence of HIV–related admissions. Finally, it was sometimes difficult to ascertain whether patients had previously received medical care elsewhere before presenting to our hospital; additionally, patients who were discharged were not followed up to track their short-term (< 30 day) and long term (≥ 30 day) clinical outcomes, which may have skewed some of our observations. Nonetheless, we believe that our study is significant because it offers insight into the morbidity and mortality patterns of hospitalized HIV-infected patients at the main referral tertiary healthcare facility in Sierra Leone and further provides on update on this important public health topic in the West Africa region.

## Conclusions

In the era of widespread ART availability globally, a high burden of HIV-related admissions and in-hospital mortality were observed at Connaught Hospital in Freetown, the main referral hospital in Sierra Leone. These findings necessitate the implementation of specific measures to enhance early HIV diagnosis and expand treatment access to all HIV-infected patients in Sierra Leone.

## Data Availability

All data and material generated or analyzed during this study are included in this manuscript. Raw de-identified study data are available from the corresponding author upon reasonable request.

## Abbreviations

AFB: Acid fast bacilli
AIDS: Acquired immune deficiency syndrome
Anti-HBc: Total hepatitis B core antibody
AOR: Adjusted odds ratio
ART: Antiretroviral therapy
CI: Confidence interval
HBsAg: Hepatitis B surface antigen
HBV: Hepatitis B virus
HCTS: HIV Counselling and Testing Services
HIV: Human immunodeficiency virus
IeDEA: International epidemiological Database to Evaluate AIDS
IQR: Interquartile range
OR: Odds ratio
PMTCT: Prevention of Mother to Child Transmission
qSOFA: Quick sequential organ failure assessment score
UNAIDS: United Nations Joint Programme on HIV and AIDS
WHO: World Health Organization
